# Universal Keyword Classifier on Public Key Based Encrypted Multikeyword Fuzzy Search in Public Cloud

**DOI:** 10.1155/2015/706102

**Published:** 2015-08-25

**Authors:** Shyamala Devi Munisamy, Arun Chokkalingam

**Affiliations:** ^1^R.M.D Engineering College, R.S.M Nagar, Kavaraipettai, Chennai, Tamil Nadu 601206, India; ^2^R.M.K College of Engineering and Technology, R.S.M Nagar, Puduvoyal, Chennai, Tamil Nadu 601206, India

## Abstract

Cloud computing has pioneered the emerging world by manifesting itself as a service through internet and facilitates third party infrastructure and applications. While customers have no visibility on how their data is stored on service provider's premises, it offers greater benefits in lowering infrastructure costs and delivering more flexibility and simplicity in managing private data. The opportunity to use cloud services on pay-per-use basis provides comfort for private data owners in managing costs and data. With the pervasive usage of internet, the focus has now shifted towards effective data utilization on the cloud without compromising security concerns. In the pursuit of increasing data utilization on public cloud storage, the key is to make effective data access through several fuzzy searching techniques. In this paper, we have discussed the existing fuzzy searching techniques and focused on reducing the searching time on the cloud storage server for effective data utilization. Our proposed *Asymmetric Classifier Multikeyword Fuzzy Search* method provides classifier search server that creates universal keyword classifier for the multiple keyword request which greatly reduces the searching time by learning the search path pattern for all the keywords in the fuzzy keyword set. The objective of using BTree fuzzy searchable index is to resolve typos and representation inconsistencies and also to facilitate effective data utilization.

## 1. Introduction

Cloud computing [1, 2] makes the infrastructure, platform, and software as a service for the worldwide users. The cloud paradigm makes the user outsource their personal data to the cloud storage [3] server which facilitates the users' access to their data anywhere at any time. The data users of the cloud storage have to pay only for the actual storage they use. Some companies will use the cloud storage for their data backup.

*Problem Formulation*. We highlight here that the cloud storage server has the responsibility to keep their customer data to be available and accessible all the time. The cloud storage must facilitate their customers to access their wide range of resources, application, and data through internet service interface immediately and fast. The number of customers utilizing the cloud storage increases significantly every day. The data on the cloud storage increases dynamically due to the increasing demands of existing customer and addition of new customers. This means the cloud storage is under a state to respond to increasing customer data and effective access to their data. To retain their customers, the cloud storage must optimize its computational time for searching the requested data. It must have some efficient searching method or additional provisions to serve their customers to provide the requested data immediately. So with this observation, we propose a new searching method named Asymmetric Classifier Multikeyword Fuzzy Search which utilizes the universal keyword classifier to store the search path pattern of all the keywords of their customers data. This allows the cloud storage server to use its time effectively to perform multiprocessing of their growing customers. Our scheme also resolves typos and representation inconsistencies since the searching is done on BTree fuzzy searchable index.

*Our Contributions*. In this paper, we propose new Scheme* Asymmetric Classifier Multikeyword Fuzzy Search (ACMFS)* which greatly reduces the time spent for searching the data and delivers the requested file immediately to the users. It also utilizes the data effectively from the cloud storage through fuzzy search on BTree fuzzy searchable index. Experimental results shows effective data utilization and the search efficiency of the proposed scheme. Our contributions are summarized as follows:

(i) Asymmetric Searchable Encryption allows the server to search over encrypted BTree fuzzy searchable index thereby providing effective data utilization.
(ii) The cloud storage server would not disclose the files to illegal access as they do not have the information about the multiple keywords *MKW* and files.
(iii) As the BTree fuzzy searchable index is created from wild card fuzzy keyword set, it tolerates typos and representation inconsistencies of authorized users.
(iv) Classifier search server uses universal keyword classifier for traversing the storage efficient BTree wild card fuzzy searchable index which stores all the search path pattern of the multiple keywords of the entire encrypted files.

*Paper Organization*. The rest of the paper is organized as follows. The related modules are discussed in Section 2 along with the limitations of the existing searching methods. In Section 3, we formulate our problem by designing the system model and goals of the proposed solution. Then we provide the detailed description of* Asymmetric Classifier Multikeyword Fuzzy Search* scheme in Section 4 followed by Section 5, which discusses the detailed design and implementation of algorithms of our proposed method. The Experimental results and performance analysis with output are shown in Section 6. We conclude our paper in Section 7.

## 2. Background

### 2.1. Related Work

Although Cloud Service Provider (CSP) hosts several third party data, Liu et al. [4] pointed out that managing sensitive data leads to security and privacy concerns. Cryptographic methods can be used to disclose the key only to authorized users to protect the data from untrusted CSP.

Ren et al. [5] state that users have several types of typing behaviour for keywords which are commonly termed as typos, representation inconsistencies, and typing habits. They suggested fuzzy keyword search to overcome these inconsistencies. Though the fuzzy keyword search is prevalent in popular search engines like Google, Bing, and so forth, it still poses risk in cloud storage due to inherent security and privacy obstacles. The searchable encryption [6–8] is recommended which takes encrypted data as files labeled with keywords and lets user securely search over the files through predefined keywords for retrieving them.

Zhou et al. [9] created k-gram based fuzzy keyword set for keywords W of the encrypted files C and Jaccard coefficient to calculate the keywords similarity.

Wang et al. [10] pointed out that keyword holds sensitive information of the files and thus keyword privacy must be protected for effective data utilization. Xu et al. [11] identified that third party could access the files by knowing the keyword search trapdoor. Xu proposed public key encryption with fuzzy keyword search (PEFKS) in which each keyword corresponds to exact keyword search trapdoor and fuzzy keyword search trapdoor.

Wang et al. [12] discusses that the search over encrypted data not only involves information retrieval techniques such as data structures for representing the searchable index but also depends on efficient search algorithms that run on the index.

### 2.2. Limitation of the Existing Methods

1. Secured and privacy preserving keyword search [4]:
  - The encryption and decryption process incurs high communication and computational cost.
2. Secured fuzzy keyword search [5]:
  - It does not support fuzzy search with public key based searchable encryption.
  - It could not carry out multiple keywords semantic search.
  - The update operation on fuzzy searchable index is not much efficient.
3. K-gram based fuzzy keyword Ranked search [9]:
  - The k-gram based fuzzy keyword set size depends on the jaccard coefficient value.
4. Verifiable fuzzy keyword search (VFKS) [10]:
  - The symbol tree fuzzy searchable index occupies more space in this search.
5. Public key encryption with fuzzy keyword search:
  - Creating fuzzy keyword index and exact keyword index is not compatible with large database.
6. Privacy-preserving multikeyword fuzzy search [12]:
  - It demands files with relatively high score to reduce the false negative rate.

## 3. Methodology of Our Scheme

### 3.1. Cloud Data Utilization Service Architecture

**Figure 1:**
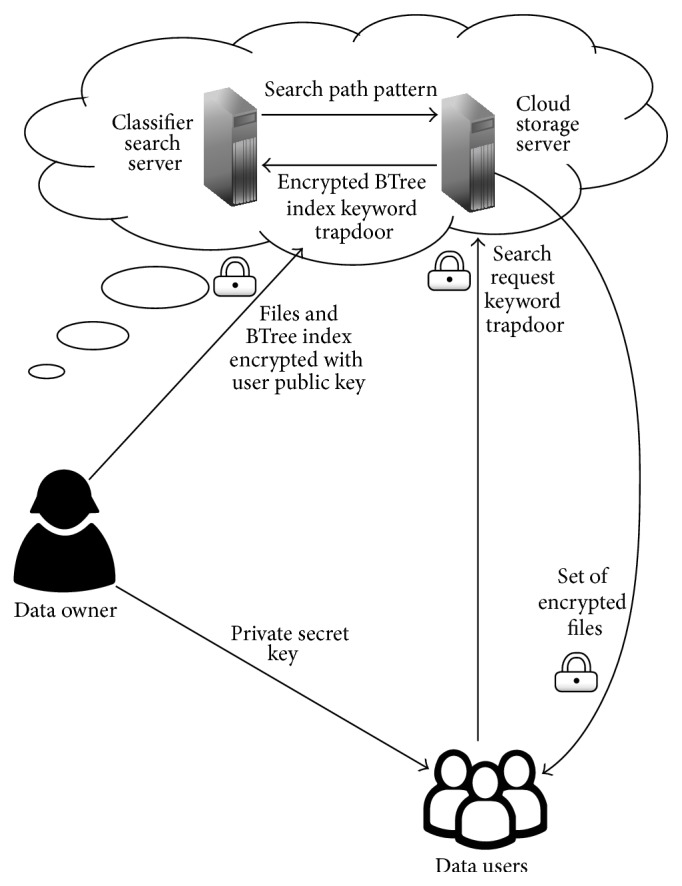
Cloud data utilization service architecture for Asymmetric Classifier Multikeyword Fuzzy Search.

In this paper, we consider our cloud data utilization service architecture which consists of four entities as data owner, cloud storage server, classifier search server, and data users and is shown in Figure 1. Here we assume that the authorization is suitably done between the data owner and data users.

Initially, the data owner generates user's public and private key pair as (*PUB*_*KEY*_, *PRIV*_*KEY*_). Data owner has a set of *K* data files *DF* = {*DF*_1_, *DF*_2_,…, *DF*_*K*_} that are encrypted with user's public key *PUB*_*KEY*_ and are outsourced to the cloud storage server. Data owner predefine multiple keywords for each file. Data owner has a set of multiple keywords *MKW* = {(*mk*_11_, *mk*_12_,…, *mk*_1*n*_), (*mk*_21_, *mk*_22_,…, *mk*_2*n*_),…, (*mk*_*k*1_, *mk*_*k*2_,…, *mk*_*kn*_)} of *K* data files. Data owner creates storage efficient wild card based fuzzy multikeyword set as *FMKS* = {(*fmk*_11_[], *fmk*_12_[],…, *fmk*_1*n*_[]), (*fmk*_21_[], *fmk*_22_[],…, *fmk*_2*n*_[]),…, (*fmk*_*k*1_[], *fmk*_*k*2_[],…, *fmk*_*kn*_[])} using wild card based technique with the predefined edit distance value. Data owner creates BTree wild card fuzzy searchable index *BSI*_*WC*_ from fuzzy multikeyword set. Data owner encrypts the files and index *BSI*_*WC*_ using user public key and is outsourced to the cloud storage server. Data owner sends the user private key as private secret key which is used by the data users for creating keyword trapdoor and for decrypting the file. Now the cloud storage server has the encrypted *K* data files DF and encrypted BTree wild card fuzzy searchable index *BSI*_*E*_. The cloud storage server shares the encrypted BTree wild card fuzzy searchable index *BSI*_*E*_ to the classifier search server. The data user requests the multiple search keywords which are encrypted using the private secret key to create multikeyword trapdoor *MKT*_*W*_ which is sent to the cloud storage server. The server sends the request *MKT*_*W*_ to the classifier search server. The universal keyword classifier receives the request *MKT*_*W*_ to check whether the request is coming for the first time. If the request is arriving for the first time; then the keyword classifier captures and stores the path of the *MKT*_*W*_ by searching over the encrypted BTree wild card fuzzy searchable index *BSI*_*E*_ and sends the search path to the cloud storage server. If the request given by the user matches a previous request then it is a repeated multiple keyword. Then the classifier search server extracts the stored search path patterns of the repeated multikeyword from the universal keyword classifier and the search path is sent to the cloud storage server. After receiving the search path pattern of the multiple keywords from the classifier search server, the cloud storage server extracts the set of encrypted files from *DF* and is sent to the data users. After receiving the encrypted files, the data user decrypts the files using private secret key.

### 3.2. Design Goals

To effectively optimize the searching time for the multiple keywords in the cloud storage server and for tolerating the typos and representation inconsistencies of authorized users, our searching method seeks to achieve the following design goals.

Search efficiency goals are

- to construct the universal keyword classifier for BTree wild card fuzzy searchable index for optimizing search time and for tolerating typos and representation inconsistencies of authorized users.

Security goals are

- to avoid the cloud storage server from getting the knowledge of data files and keyword set. This is achieved by outsourcing the encrypted files and index to the cloud storage server.

Privacy goals are

- to provide user privacy by abstracting the details of data files, keyword, and index to the cloud storage server;
- to support data privacy by encrypting the files and index with user public key before outsourcing to the cloud storage server;
- to attain keyword privacy by forming BTree wild card fuzzy searchable index from the fuzzy multikeyword set for the predefined set of multiple keywords;
- to achieve query privacy by sending k-gram keyword trapdoor encrypted with the private secret key;
- to accomplish index privacy by creating encrypted BTree wild card fuzzy searchable index.

## 4. Asymmetric Classifier Multikeyword Fuzzy Search (ACMFS)

### 4.1. Notations and Preliminaries

They are as follows.

(i) **P** **S** **K**: private secret key;
(ii) **edit**: edit distance;
(iii) **D** **F** = {**D** **F**_1_, **D** **F**_2_,…, **D** **F**_**K**_}: set of *K* encrypted data files *DF*;
(iv) **P** **U** **B**_**KEY**_: user public key;
(v) **O** **P** **K**_**PUB**_: owner public key;
(vi) **P** **R** **I** **V**_**KEY**_: user private key;
(vii) **M** **K** **W** = {(**m** **k**_11_, **m** **k**_12_,…, **m** **k**_1**n**_), (**m** **k**_21_, **m** **k**_22_,…, **m** **k**_2**n**_),…, (**m** **k**_**k**1_, **m** **k**_**k**2_,…, **m** **k**_**kn**_)}: predefined multiple keywords set of DF;
(viii) **F** **M** **K** **S** = {(**f** **m** **k**_11_[], **f** **m** **k**_12_[],…, **f** **m** **k**_1**n**_[]),…, (**f** **m** **k**_**k**1_[], **f** **m** **k**_**k**2_[],…, **f** **m** **k**_**kn**_[])}: fuzzy multikeyword set;
(ix) **B** **S** **I**_**WC**_: BTree wild card fuzzy searchable index;
(x) **B** **S** **I**_**E**_: encrypted BTree wild card fuzzy searchable index of *BSI*_*WC*_;
(xi) **S** **P** **P**: search path pattern of multiple keywords;
(xii) **M** **K** **T**_**W**_: encrypted multikeyword trapdoor search request;
(xiii) **M** **F** **I** **L** **E**_**ENC**_: set of encrypted multiple files matching search request;
(xiv) **M** **F** **I** **L** **E**[]: decrypted multiple files of MFILE_ENC_;
(xv) **M** **S** **R**: multikeyword search request.

### 4.2. Searchable Encryption

Searchable encryption [13] is a cryptographic technique where the data users search the encrypted searchable index by the following steps.

- The encrypted tokens in the searchable index have the pointers to encrypted files. Token symbols are the encrypted keyword.
- If the requested token found a match in the searchable index, then it extracts the file pointer without decryption.
- If the token is not found in the searchable index, then it returns the null file pointer.

The two types of searchable encryption are symmetric and asymmetric (public key based) searchable encryption [14, 15]. In Symmetric Searchable Encryption, the data owner who outsources the encrypted index and data and the server that searches the data share the same secret keys. The efficiency of SSE is high since it uses symmetric cryptographic methods such as block ciphers, pseudorandom functions, and hash functions. The disadvantage of SSE is that the server has high probability to learn about the owner data and keywords. In Asymmetric Searchable Encryption [16, 17], the data owner outsources the index that is encrypted by the user's public key. The keyword trapdoor is created by the user's private key. So only the authorized users can request the search from the server. The advantage of ASE is that it supports conjunctive or disjunctive keywords searches. The disadvantage of SSE is that it suffers from KGA.

### 4.3. Edit Distance

Edit distance is the method of quantifying the similarity of the two strings. The edit distance (*S*_1_, *S*_2_) between two strings *S*_1_ and *S*_2_ is the smallest number of operations necessary to change one string to another. The three primitive operations are as follows:

- insertion: inserting one character into the string;
- deletion: deleting one character from the string;
- substitution: changing one character to another in the string.

The edit distance of the two strings in our system is analysed by dynamic programming. By dynamic programming strategy, the edit distance ed(*x*, *y*) of any two strings “*x*” and “*y*” is defined as follows and refer to Algorithm 1.

 Assume the strings are *x*[0,1,…, *i* − 1] and *y*[0,1,…, *j* − 1].
 If *x*, *y* = 0, then ed(*x*, *y*) = 0.
 If *y* = 0, then ed(*x*, 0) = *i*.
 If *x* = 0, then ed(0, *y*) = *j*.
 If *x* ≠ *y*, then ed(*x*, *y*) = min{ed(*i* − 1, *j*) + 1, ed(*i*, *j* − 1) + 1, ed(*i* − 1, *j* − 1) + diff(*x*[*i* − 1], *y*[*j* − 1])}.
 Note that if *x* = *y* then diff(*x*, *y*) = 0 else diff(*x*, *y*) = 1.

Examples for ed(*S*_1_, *S*_2_) = 1 are as follows:

 insertion: *S*_1_ = “SAMI” and *S*_2_ = “SAMIY”;
 deletion: *S*_1_ = “SAMI” and *S*_2_ = “SAM”;
 substitution: *S*_1_ = “SAMI” and *S*_2_ = “SAME”.

### 4.4. Fuzzy Keyword Set

Since different users have various typing behaviors, they may misspell the keywords. So the fuzzy keyword set is formed to effectively utilize the data. Fuzzy keyword set can be created by wild card based technique, k-gram based technique, and symbol tree based technique. For example, fuzzy keyword set for “W = HEN” with edit distance = 1 is as follows:

 FKS_ed=1_ = {(A ⋯ Z)HEN, H(A ⋯ Z)EN, HE(A ⋯ Z)N, HEN(A ⋯ Z), EN, HN, HE, (A ⋯ Z)EN, H(A ⋯ Z)N, HE(A ⋯ Z), HEN}.

Total number of keywords = 186.

### 4.5. Fuzzy Keyword Search

For the set of *M* data files *D* = {*D*_1_, *D*_2_,…, *D*_*M*_} with the predefined set of *KW* = {*kw*_1_, *kw*_2_,…, *Kw*_*n*_}, the fuzzy keyword search fuzzy (w, FKS_ed=1_) is as shown in Algorithm 2.

### 4.6. Wild Card Based Technique

Wild card based technique is used to create storage efficient wild card based fuzzy keyword set. We use the wild card “#” character to represent the positions of three edit distance operations such as insertion, deletion, and substitution thereby creating tiny fuzzy keyword set. For example, the wild card based fuzzy keyword set for w = “HEN” with edit distance ed = 1 is FKS_HEN,1_ as follows:

 FKS_HEN,1_ = {#HEN, H#EN, HE#N, HEN#, EN, HN, HE, #EN, H#N, HE#, HEN}.

Total number of keywords = 11.

The length of fuzzy keyword set is **L** = ((2**n** + 1)*∗*26) + **n** + 1. The length of wild card based fuzzy keyword set is **L** = (2**n** + 1) + **n** + 1.

### 4.7. K-Gram Based Technique

K-gram based technique is used to create k-gram based fuzzy keyword set for the predefined gram value k. The keywords of k-gram based fuzzy keyword set is the subset of keyword set. For example, the k-gram based fuzzy keyword set for w = “HEN” with gram value k = 1 is FKS_K=1_ as follows:

 FKS_K=1_ = {KY, SY, KY}.

Total number of keywords = 3.

### 4.8. Assumptions of ADKEFS

Before we start our framework design, we have the following assumptions on our proposed scheme Asymmetric Classifier Multikeyword Fuzzy Search* ACMFS*.

- We assume that the cloud storage server concentrates on servicing more customers and not to leave partnership of their customers from the business.
- Here we assume that the authorization is suitably done between the data owner and data users.
- Data owner creates users private and public key pair.
- We assume the wild card based fuzzy multikeyword set FMKS of multikeyword set MKW contains the original keyword as the first component.
- We assume that each file has multiple keywords and it is possible for a keyword to be the same for multiple files.

## 5. Implementation of Asymmetric Classifier Multikeyword Fuzzy Search (ACMFS)

Here we discuss our proposed scheme in detail with algorithm for all the functions involved.

### 5.1. Function Definitions of ADKEFS

The following functions are implemented to optimize the searching on the cloud storage server and to achieve the effective data utilization.

Functions on data owner are

(i) CreateKeyPairsForUser(SECRET_1_, SECRET_2_),
(ii) EncryptMultiKeywordDataFile(*PUB*_*KEY*_[]),
(iii) CreateWildCardFuzzyMultiKeywordSet(MKW[][], edit),
(iv) CreateBTreeWildcardFuzzySearchableIndex(FMKS[][][]),
(v) EncryptBTreeWildcardFuzzySearchableIndex(*BSI*_*WC*_,* PUB*_*KEY*_[]).

Functions on cloud storage server are

(i) ExtractMultipleFileUsingPattern(SPP[]).

Functions on classifier search server are

(i) SearchBTreeWildCardFuzzySearchableIndex(BSI_E_, MKT_*W*_[][]).

Functions on data user are

(i) ViewDecryptedMultiKeywordFile(*PRIV*_*KEY*_[], MFILE_ENC_).

### 5.2. Overall Framework of Asymmetric Classifier Multikeyword Fuzzy Search (ACMFS)

**Figure 2:**
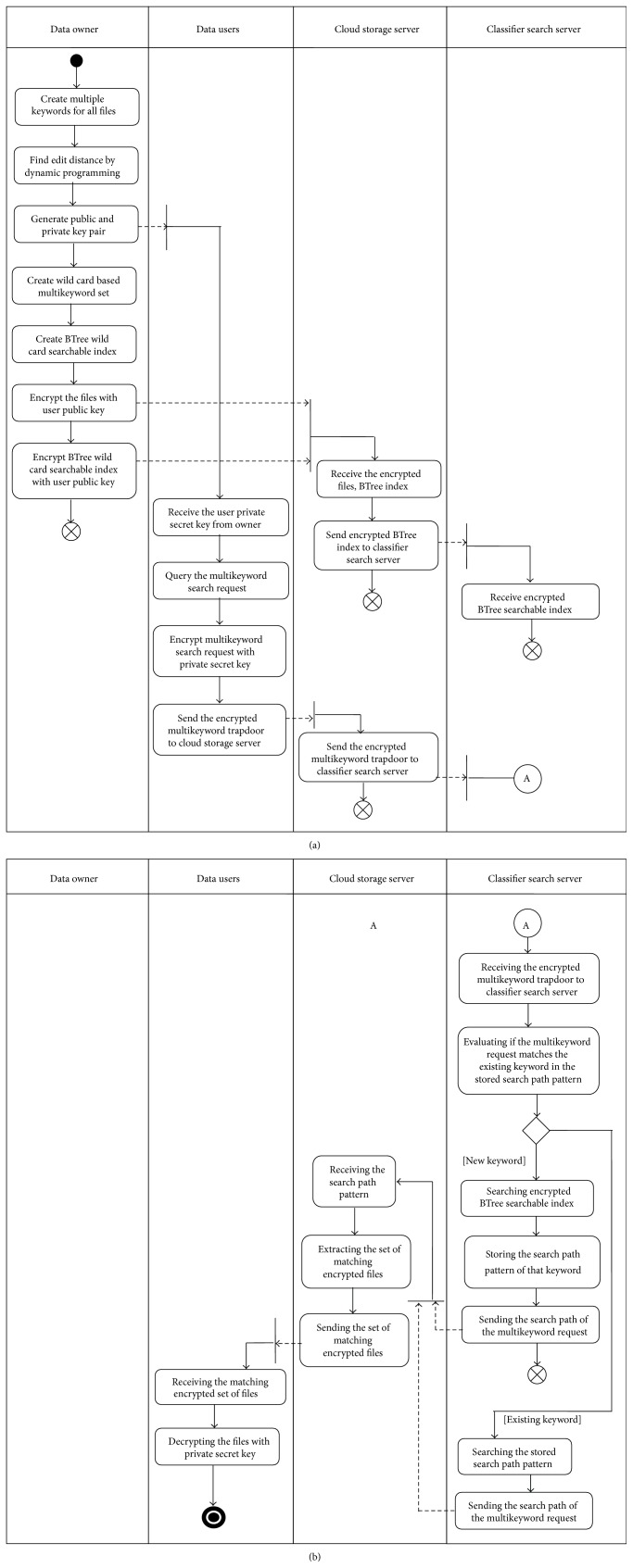
Activity diagram for Asymmetric Classifier Multikeyword Fuzzy Search.

Our proposed method Asymmetric Classifier Multikeyword Fuzzy Search has classifier search server that creates the search path pattern for all the keywords of the encrypted set of *K* data files. Data owner creates encrypted BTree wild card fuzzy searchable index for the fuzzy multikeyword set and is outsourced to the cloud storage server. This overcomes the problem of typos and representation inconsistencies behaviour of the data users. The overall conceptual description of Asymmetric Classifier Multikeyword Fuzzy Search is shown in Figure 2 as an activity diagram. Please refer to Algorithm 3 for the pseudo-code.

### 5.3. Key Generation Algorithm

Here we use RSA public key algorithm for generating public and private key. Here this takes two secret keys SECRET_1_ and SECRET_2_ which is predefined by the data owner where both secret keys SECRET_1_, SECRET_2_ = {0,1}^*∗*^.  Algorithm 4 is executed by the data owner to generate public and private key pair. Data owner sends the user private key as private secret key which is used by the data users to create keyword trapdoor and for decrypting the file.

### 5.4. File Encryption Algorithm

Data owner executes Algorithm 5 to form encrypted set of *K* data files *DF* = {*DF*_1_, *DF*_2_,…, *DF*_*K*_} that are encrypted with user's public key* PUB*_*KEY*_ and are outsourced to the cloud storage server.

### 5.5. File Decryption Algorithm

After receiving the search path pattern of the multiple keywords from the classifier search server, the cloud storage server extracts the set of encrypted files from* DF* and is sent to the data users. After receiving the encrypted files, the data user decrypts the files using private secret key by executing Algorithm 6.

### 5.6. Wild Card Based Fuzzy Multikeyword Set Algorithm

Data owner has MKW = {(mk11, mk12,…, mk1n), (mk21, mk22,…, mk2n),…, (mkn1, mkn2,…, mkkn)} a set of multiple keywords of *K* data files. Data owner creates storage efficient fuzzy multikeyword set FMKS = {(fmk11[], fmk12[],…, fmk1n[]), (fmk21[], fmk22[],…, fmk2n[]),…, (fmkk1[], fmkk2[],…, fmkkn[])} using wild card based technique with the predefined edit distance value. Data owner executes Algorithm 7 to form fuzzy multikeyword Set.

### 5.7. BTree Wild Card Fuzzy Searchable Index Algorithm

Data owner creates BTree wild card fuzzy searchable index BSIWC from fuzzy multikeyword set. Data owner executes Algorithm 8 to create BTree wild card fuzzy searchable index BSIWC for the wild card based fuzzy keyword set FKS.

### 5.8. Encrypting BTree Fuzzy Searchable Index Algorithm

Data owner encrypts the BTree wild card fuzzy searchable index BSIWC using user public key and is outsourced to the cloud storage by executing Algorithm 9 to create encrypted BTree wild card fuzzy searchable index BSIE and is outsourced to the cloud storage server.

### 5.9. Searching Encrypted BTree Fuzzy Searchable Index Algorithm

The data user encrypts the multiple search keywords using the private secret key to create multikeyword trapdoor MKTW to the cloud storage server. The server sends the request MKTW to the classifier search server. The universal keyword classifier receives the request MKTW to check whether the request is coming for the first time. If the request is arriving for the first time, then the keyword classifier captures and stores the path of the MKTW by searching over the encrypted BTree wild card fuzzy searchable index BSIE by executing Algorithm 10 and sends the search path to the cloud storage server. If the request given by the user matches a previous request stored then it is a repeated multiple keyword. Then the classifier search server extracts the stored search path patterns of the repeated multikeyword from the universal keyword classifier and the search path is sent to the cloud storage server.

## 6. Implementation Results

### 6.1. Implementation Setup

The implementation of the proposed work was accomplished through Asymmetric Classifier Multikeyword Fuzzy Search (ACMFS) cloud data utilization service architecture using Jelastic PaaS LayerShift cloud storage provider which offers infrastructure, platform, and application as a service for the customers. The experimentation was carried out with the code programmed in JAVA for data owner, users, classifier search server, and cloud storage server. Microsoft SQL MYSQL 5.5.42 was enabled to act as the database for the proposed system. The simulation was performed with the setup of data owner, data users from our side, and classifier search server, cloud storage server on the Jelastic cloud storage. The data owner authenticates 100 users and defines the multikeyword set for each data files. Prior to evaluating the results, the data owner outsourced 1000 encrypted files to the Jelastic cloud storage. The data owner creates the wild card fuzzy multikeyword set FMKS for edit distance 1, 2 and BTree fuzzy searchable index BSI_WC_. The data owner outsources the encrypted BSI_WC_ and 1000 encrypted files to Jelastic cloud storage server. The cloud storage now contains the encrypted 1000 files and encrypted BSI_WC_. With this simulated setup, the authorized users are allowed to access the files in the cloud storage using their individual identity. The users are now allowed to access the files in the cloud storage by entering the multiple keyword search request.

### 6.2. Performance Analysis

**Figure 3:**
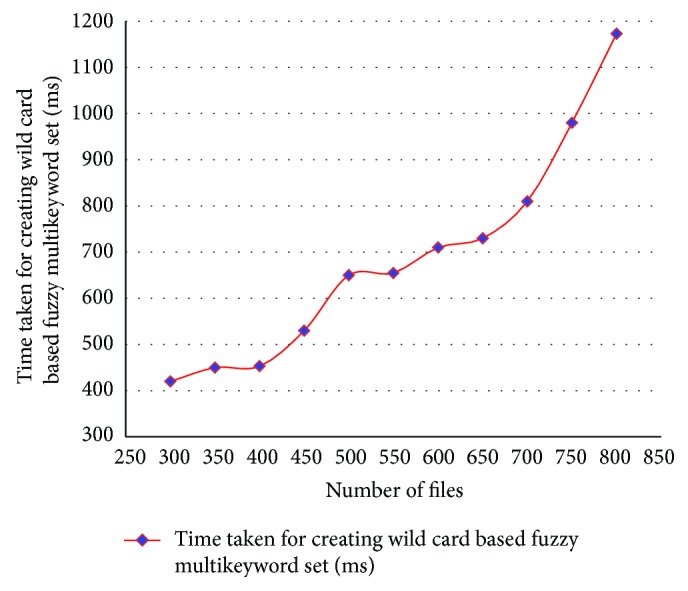
Time taken for creating wild card fuzzy multikeyword set for ACMFS.

**Figure 4:**
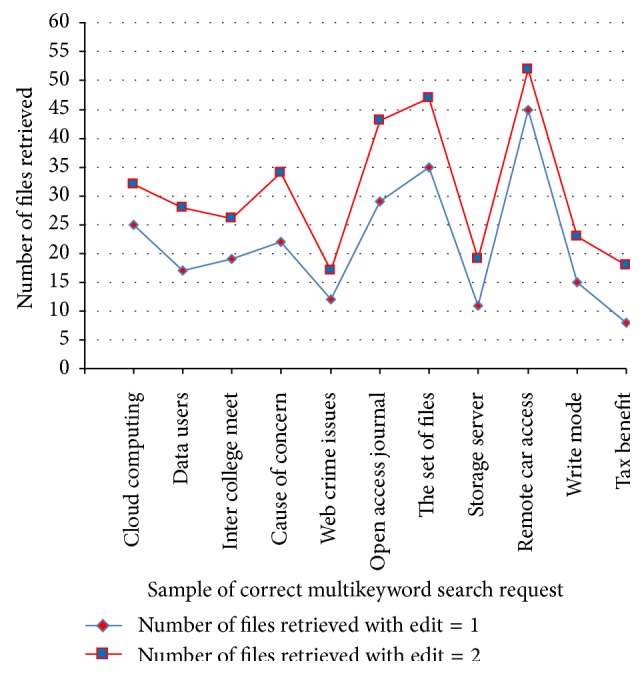
Data utilization efficiency for correct keywords in ACMFS.

**Figure 5:**
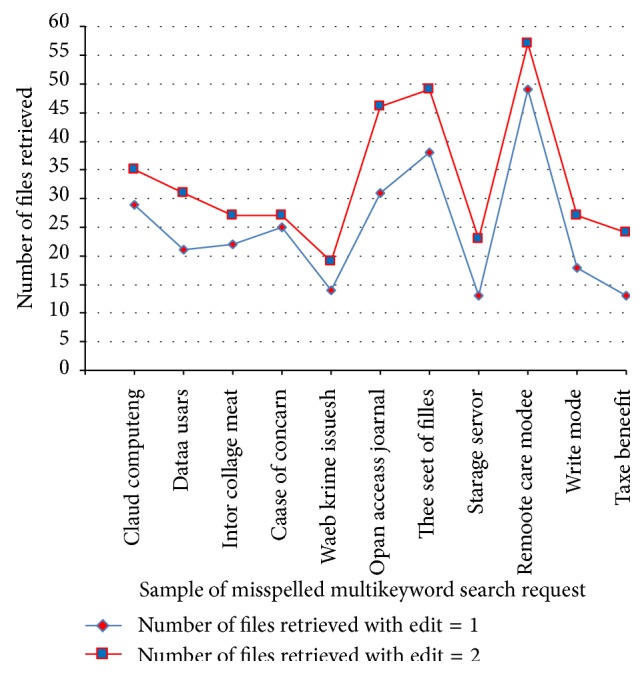
Data utilization efficiency for misspelled keywords in ACMFS.

**Figure 6:**
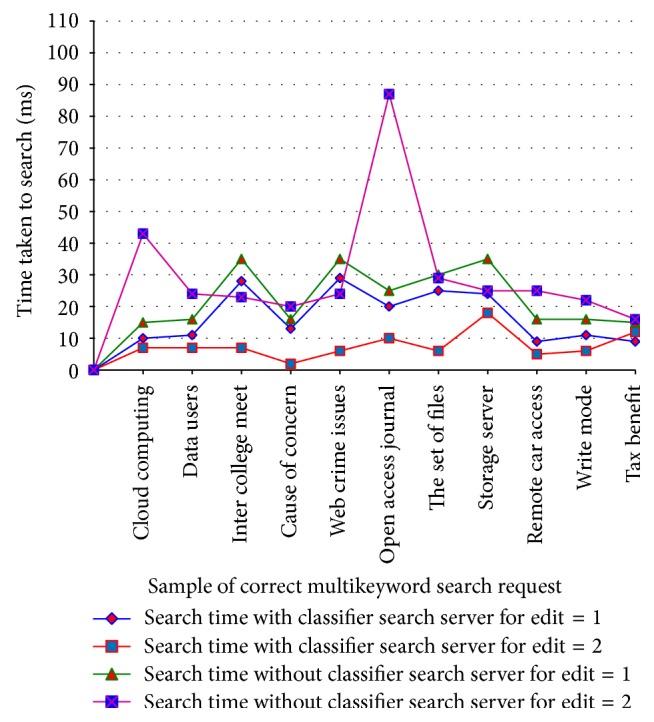
Search efficiency for correct keywords in ACMFS.

**Figure 7:**
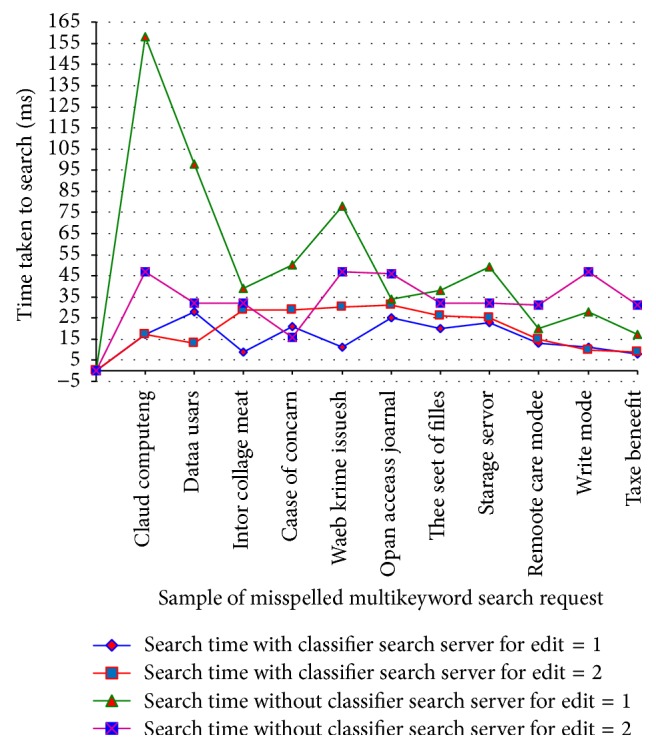
Search efficiency for misspelled keywords in ACMFS.

The performance of the proposed method was evaluated taking into account the search time efficiency and data utilization from the Jelastic cloud storage by giving the multiple keyword search request from the data users with classifier search server. The experimental results obtained by ACMFS cloud data utilization system architecture are shown in Tables 1–5 and Figures 3–7.  Table 1 shows the analysis of time taken for creating the wild card based fuzzy multikeyword set with different number of users and files and its analysis chart is shown in Figure 3. Here data owner predefines five keywords for each file.

Table 2 shows the analysis of data utilization efficiency for correct keyword in terms of number of files retrieved from the cloud storage and its analysis chart is shown in Figure 4.

Table 3 shows the analysis of data utilization efficiency for misspelled keyword in terms of number of files retrieved from the cloud storage and its analysis chart is shown in Figure 5.

Table 4 shows the analysis of search time efficiency for correct multikeywords with and without classifier search server for edit 1, 2 and its analysis chart is shown in Figure 6.

Table 5 shows the analysis of search time efficiency for misspelled multikeywords with and without classifier search server for edit 1, 2 and its analysis chart is shown in Figure 7.

## 7. Conclusion

This work Asymmetric Classifier Multikeyword Fuzzy Search presented a method that can be successfully used to enhance data utilization and improved search efficiency for public cloud storage. Provided that the data owner stores the set of encrypted files in cloud storage, we showed that the user experience improved search time due to the presence of classifier search server which searches the BTree wild card fuzzy searchable index. The proposed system extracts the wild card fuzzy multikeyword set for the multiple keyword search request resulting in increased data utilization in terms of number of files retrieved for the corresponding users. The proposed system's performance is demonstrated with the advent of classifier search server which stores the pattern of search and helps reducing the search time for repeated multiple keyword search request. The classifier search server concept adds a new paradigm to cloud storage server serving several thousands of data owners and their users.

## Figures and Tables

**Algorithm 1:**
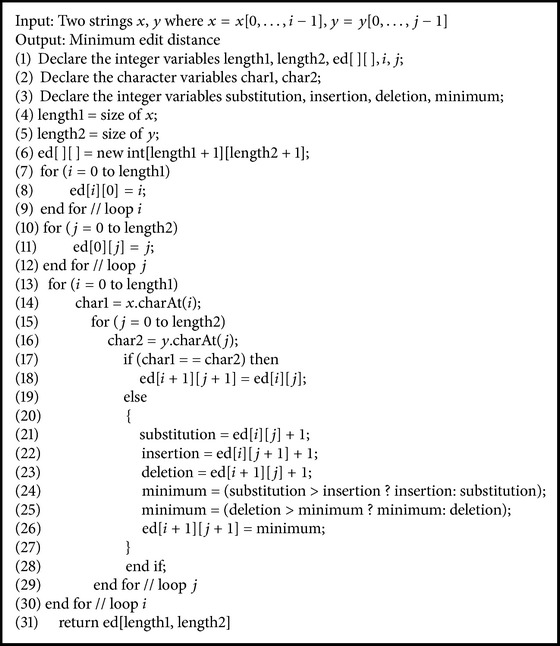
EditDistance(*x*[0,…, *i* − 1], *y*[0,…, *j* − 1]).

**Algorithm 2:**
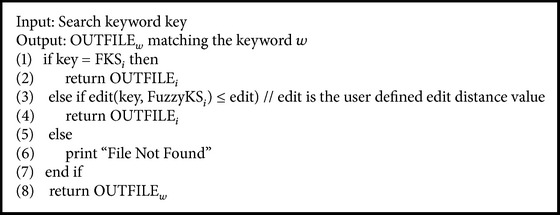
Fuzzy(key, FuzzyKS_ed=1_).

**Algorithm 3:**
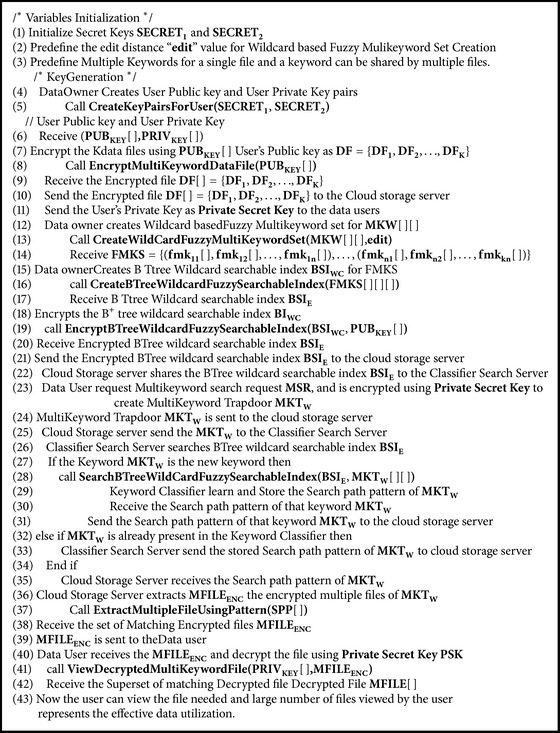
ACMFS( ) (Asymmetric Classifier Multikeyword Fuzzy Search).

**Algorithm 4:**
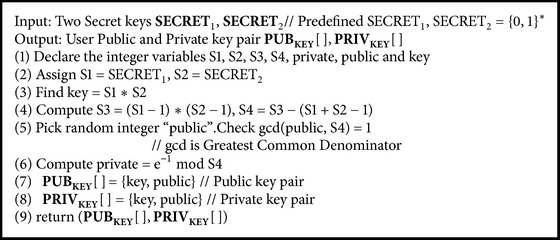
CreateKeyPairsForUser(SECRET_1_, SECRET_2_)* // Key Generation Algorithm.*

**Algorithm 5:**
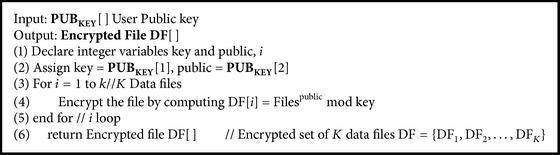
EncryptMultiKeywordDataFile(*PUB*_*KEY*_[ ]) // File Encryption Algorithm.

**Algorithm 6:**
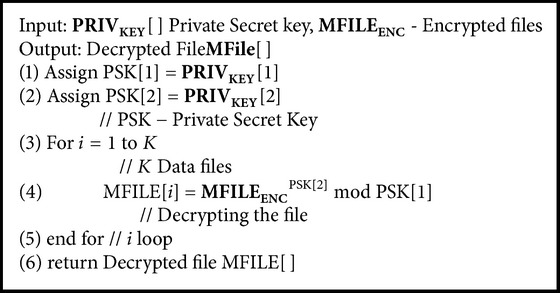
*ViewDecryptedMultiKeywordFile*(*PRIV*_*KEY*_[ ], MFILE_ENC_) // File Decryption Algorithm.

**Algorithm 7:**
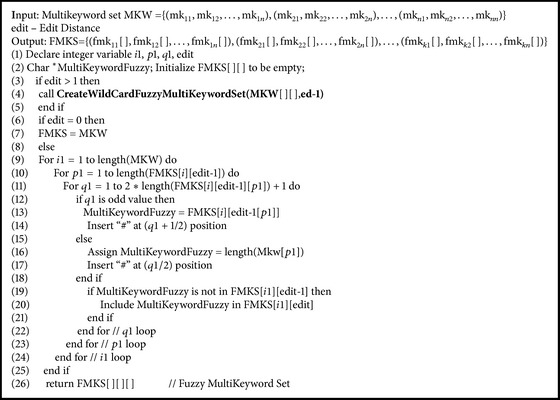
CreateWildCardFuzzyMultiKeywordSet(MKW[ ][ ],edit).

**Algorithm 8:**
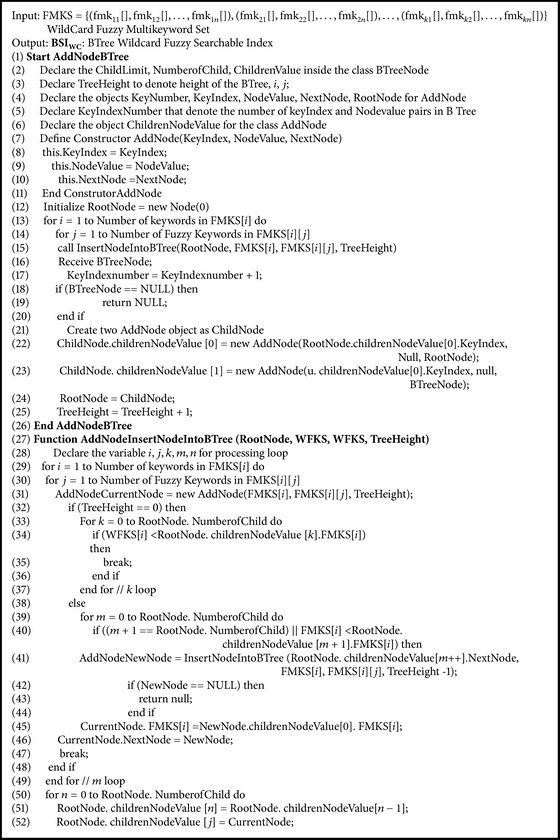
CreateBTreeWildCardFuzzySearchableIndex(FMKS[][][]).

**Algorithm 9:**
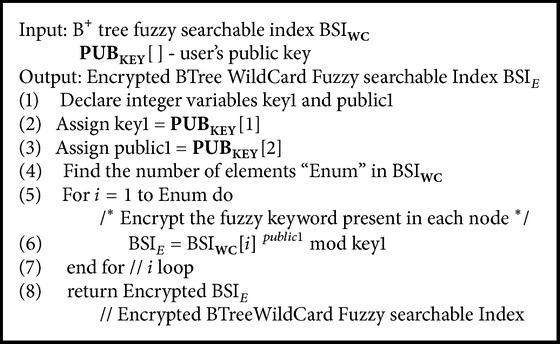
EncryptBTreeWildcardFuzzySearchableIndex(BSI_WC_, PUB_KEY_[ ]).

**Algorithm 10:**
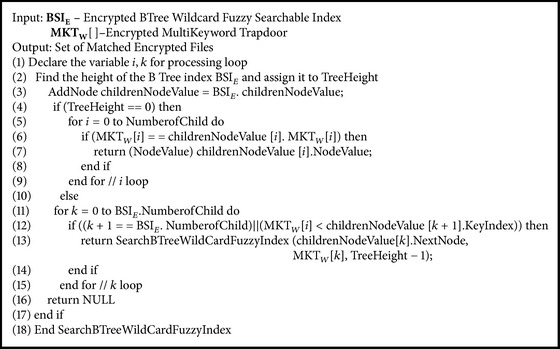
SearchBTreeWildCardFuzzySearchableIndex(BSI_E_, MKT_*W*_[ ][ ]).

**Table 1:** Analysis of time taken for creating the wild card based fuzzy multikeyword set.

Number of users	Number of files	Time taken for creating wild card based fuzzy multikeyword set (ms)
10	300	420
12	350	450
14	400	453
16	450	530
18	500	650
20	550	655
22	600	710
24	650	730
26	700	810
28	750	980
30	800	1173

**Table 2:** Analysis of data utilization efficiency for correct keywords.

Number of users	Number of files	Sample of correct multikeyword search request	Number of files retrieved with edit = 1	Number of files retrieved with edit = 2
10	300	Cloud computing	25	32
12	350	Data users	17	28
14	400	Inter college meet	19	26
16	450	Cause of concern	22	34
18	500	Web crime issues	12	17
20	550	Open access journal	29	43
22	600	The set of files	35	47
24	650	Storage server	11	19
26	700	Remote car access	45	52
28	750	Write mode	15	23
30	800	Tax benefit	8	18

**Table 3:** Analysis of data utilization efficiency for misspelled keywords.

Number of users	Number of files	Sample of misspelled multikeyword search request	Number of files retrieved with edit = 1	Number of files retrieved with edit = 2
10	300	Claud computeng	29	35
12	350	Dataa usars	21	31
14	400	Intor collage meat	22	27
16	450	Caase of concarn	25	27
18	500	Waeb krime issuesh	14	19
20	550	Opan acceass joarnal	31	46
22	600	Thee seet of filles	38	49
24	650	Starage servor	13	23
26	700	Remoote care accesh	49	57
28	750	Writee modee	18	27
30	800	Taxe beneefit	13	24

**Table 4:** Analysis of search efficiency for correct keywords with and without classifier search server.

Number of users	Number of files	Sample of correct multikeyword search request	Search time with classifier search server for edit = 1 (ms)	Search time with classifier search server for edit = 2 (ms)	Search time without classifier search server for edit = 1 (ms)	Search time without classifier search server for edit = 2 (ms)
10	300	Cloud computing	10	7	15	43
12	350	Data users	11	7	16	24
14	400	Inter college meet	28	7	35	23
16	450	Cause of concern	13	2	16	20
18	500	Web crime issues	29	6	35	24
20	550	Open access journal	20	10	25	87
22	600	The set of files	25	6	30	29
24	650	Storage server	24	18	35	25
26	700	Remote car access	9	5	16	25
28	750	Write mode	11	6	16	22
30	800	Tax benefit	9	12	15	16

**Table 5:** Analysis of search efficiency for misspelled keywords with and without classifier search server.

Number of users	Number of files	Sample of misspelled multikeyword search request	Search time with classifier search server for edit = 1	Search time with classifier search server for edit = 2	Search time without classifier search server for edit = 1	Search time without classifier search server for edit = 2
10	300	Claud computeng	17	17	158	47
12	350	Dataa usars	28	13	98	32
14	400	Intor collage meat	9	29	39	32
16	450	Caase of concarn	21	29	50	16
18	500	Waeb krime issuesh	11	30	78	47
20	550	Opan acceass joarnal	25	31	34	46
22	600	Thee seet of filles	20	26	38	32
24	650	Starage servor	23	25	49	32
26	700	Remoote care accesh	13	15	20	31
28	750	Writee modee	11	10	28	47
30	800	Taxe beneefit	8	9	17	31

## Appendix

See Algorithms 1 and 3–10.
